# The albumin-exendin-4 recombinant protein E2HSA improves glycemic control and β-cell function in spontaneous diabetic KKAy mice

**DOI:** 10.1186/s40360-017-0143-8

**Published:** 2017-06-19

**Authors:** Caina Li, Shaocong Hou, Shuainan Liu, Yi Huan, Sujuan Sun, Quan Liu, Zhufang Shen

**Affiliations:** State Key Laboratory of Bioactive Substances and Functions of Natural Medicines, Institute of Materia Medica, Chinese Academy of Medical Sciences and Peking Union Medical College, Xiannongtan Street, Beijing, 100050 China

**Keywords:** E2HSA, Type 2 diabetes, β-cell function, Hyperglycemia, Dyslipidemia

## Abstract

**Background:**

E2HSA is a genetic fusion protein that consists of two tandem exendin-4 molecules that are covalently bonded to recombinant human serum albumin via a peptide linker. Previous studies have demonstrated that E2HSA significantly decreased blood glucose levels, improved β-cell function and promoted β-cell proliferation in diabetic db/dB mice. This study aimed to evaluate the benefits of E2HSA on glucose and lipid metabolism in a spontaneous diabetes animal model, KKAy mice.

**Methods:**

E2HSA was acutely administered at doses of 1, 3 and 9 mg/kg by subcutaneous injection in diabetic KKAy mice with exendin-4 (2 μg/kg) as a positive reference, and then the non-fasting blood glucose and food intake levels were dynamically monitored. In addition, different doses of E2HSA were injected once daily, as well as with exendin-4 twice daily, for 7 weeks to evaluate the effect on glucose and lipid metabolism, as well as the body weight, food and water intake.

**Results:**

Single injection of E2HSA decreased non-fasting blood glucose and food intake levels in a dose-dependent manner for 4 days and 2 days, respectively. Repeated injections with E2HSA significantly decreased variations in blood glucose levels with a reduction of HbA1c levels by 1.6% at a 9 mg/kg dose, simultaneously increased fasting blood insulin levels, inhibited fasting blood glucagon levels, improved the impaired oral glucose tolerance and enhanced glucose infusion rate, which is the gold standard for evaluating β-cell function. Moreover, repeated injections with E2HSA also ameliorated the dyslipidemia and reduced body weight, food and water intake in diabetic KKAy mice.

**Conclusions:**

E2HSA significantly reduced blood glucose levels over a prolonged duration, enhanced β-cell function, and ameliorated dyslipidemia and obesity in diabetic KKAy mice. Thus, E2HSA may be a new candidate for the treatment of type 2 diabetes.

**Electronic supplementary material:**

The online version of this article (doi:10.1186/s40360-017-0143-8) contains supplementary material, which is available to authorized users.

## Background

Type 2 diabetes mellitus (T2DM) has become a global epidemic disease that is associated with both increased economic and clinical burdens, which also causes high mortality rates due to related micro- and macro-vascular complications, such as cardiovascular disease, kidney failure, amputations and blindness. Large fluctuations in blood glucose levels has been identified as the biggest culprit in causing diabetic complications, and strictly restraining blood glucose levels by keeping glycated hemoglobin (HbA1c) levels < 7.0% has been the main outcome for diabetes treatment [1]. Physicians have a variety of choices for individualizing treatment and use numerous available drugs, including metformin, sulfonylureas, thiazolidinediones, insulin, etc. Unfortunately, frequent hypoglycemia and weight gain, as well as poor compliance of insulin injection, reduces positive health outcomes in patients.

Glucagon like-peptide-1 (GLP-1) is an endocrine hormone that is secreted by intestinal L cells in response to nutrient stimulation. GLP-1 performs a hypoglycemic function by simultaneously enhancing insulin secretion and inhibiting glucagon secretion. Unlike the aforementioned anti-diabetic drugs, GLP-1 has an advantage in inducing weight loss and only rarely causing hypoglycemia [2]. Importantly, GLP-1 provides evident protection for pancreatic islet β cells that suppresses or delays progressive failure and ultimately improves the homeostasis of glucose metabolism [3]. Liraglutide, a GLP-1 analog, has been reported to increase β-cell mass by directly regulating cell kinetics and suppressing both oxidative and endoplasmic reticulum stresses [4].

Nevertheless, GLP-1 itself is not appropriate for clinical use, having a short half-life (1–2 min) due to rapid degradation by dipeptidyl peptidase-4 and neutral endopeptidase, as well as glomerular filtration. Exendin-4 is the first approved GLP-1-based drug that must be injected twice daily [5]. E2HSA is a genetic fusion protein consisting of two tandem exendin-4 molecules that are covalently bonded to recombinant human serum albumin via a peptide linker, such as to decrease degradation and glomerular filtration [6]. This modification has been also adopted for use in other analogs, including two approved drugs, albiglutide and dulaglutide [7, 8]. Our previous studies demonstrated that E2HSA retained biological activity of exendin-4 in vitro, displayed prolonged hypoglycemic action and improved β-cell function in both normal ICR and spontaneous diabetic db/db mice [9]. This study aimed to evaluate effects of E2HSA on the duration of hypoglycemic action, control of glycemia and amelioration of β-cell function, as well as effects on lipid metabolism and body weight in spontaneous diabetic KKAy mice.

## Methods

### Design of study

A single subcutaneous injection was utilized to evaluate the hypoglycemic effect and duration, while repeated injections were used to assess effects on controlling blood glucose levels, insulin secretion, oral glucose tolerance and β-cell glucose sensitivity, as well as influences on blood lipid levels, body weight, food consumption and water intake.

### Animals

10–12 week-old female spontaneous diabetic KKAy mice (HFK bioscience Co. Ltd. Beijing, China) were housed in a temperature- and humidity-controlled environment and fed a high fat diet that consists of 78.8% basic feed, 1% cholesterol, 10% yolk powder, 10% lard and 0.2% bile salt (HFK bioscience Co. Ltd. Beijing, China) with free access to water until hyperglycemia and insulin resistance were noted. All animals were handled in accordance with the standards for laboratory animals (GB14925-2001) and guidelines for the humane treatment of laboratory animals (MOST 2006a) established by the People’s Republic of China.

### Materials

E2HSA and exendin-4 were provided by Huayang Pharmaceutical Co., Ltd. (Zhejiang, China). The insulin (mouse) ultrasensitive ELISA kit was obtained from ALPCO (America) and the glucagon (mouse) ELISA kit was from R&D (America). The HbA1c level detection kit was bought from HOMA Biological (China). Both the detection kits of triglyceride (TG) and total cholesterol (TC) were purchased from BioSino (China) and the free fatty acid (FFA) assay kit was from Sekisui Medical Co., Ltd. (Japan). Pertinent equipment used was a microplate reader (BioTek, America), a peristaltic pump (ISMATEC, Switzerland) and a glucose meter (ACCU-CHEK Active, Germany).

### Single injection of E2HSA in diabetic KKAy mice

The spontaneous diabetic KKAy mice were grouped based on non-fasting blood glucose, fasting blood glucose and body weight, and allowed diet and water ad libitum (*n* = 10). E2HSA was supplemented at doses of 1, 3 and 9 mg/kg by subcutaneous injection, while the non-fasting blood was collected from tail tips immediately 5 h later and at 9:00 am on the following days. Then the blood glucose levels were determined with glucose oxidase method. The food intake of each group was recorded every day. Exendin-4 was supplemented at dose of 2 μg/kg as the positive reference, and saline was injected as the negative control. In order to conveniently observe the effects of E2HSA, the non-fasting blood glucose was treated as normalized blood glucose by defining the blood glucose from the control group as 100% that was calculated by [100 - (BG_Con average_ - BG_Con_)/BG_Con average_ × 100], and the blood glucose from the treatment group was calculated by [100 - (BG_Con average_ - BG_Treatment_)/BG_Con average_ × 100]. BG_Con average_ is the average blood glucose of the control group (Con). BG_Con_ is the blood glucose from each mouse in the control group, while BG_treatment_ is that in the treatment group [9, 10].

### Repeated injections with E2HSA in diabetic KKAy mice

The spontaneous diabetic KKAy mice were grouped based on the fasting blood glucose, TG, TC and body weight (*n* = 13). E2HSA was subcutaneously injected at doses of 1, 3 and 9 mg/kg once daily for 7 weeks. Exendin-4 at a 2 μg/kg dose was adopted as positive reference and injected twice daily according to the manufacturer’s instructions. The non-fasting blood glucose levels at 9:00 am with food and water ad libitum, and fasting blood glucose levels after a 4 h fasting with free access to water were dynamically determined every week. The HbA1c levels were evaluated 6 weeks after treatment with food and water ad libitum. The fasting blood insulin and glucagon levels were separately measured 2 and 7 weeks after treatment with only food removed for 4 h. In addition, the blood TG, TC and FFA levels were measured at 1 and 3 weeks after treatment following a 4 h fasting with water ad libitum. The food intake, water consumption and body weight were dynamically recorded every week during the treatment.

### Oral glucose tolerance test

The oral glucose tolerance tests were performed at 1 and 4 weeks after treatment. All mice were fasted for 4 h with water ad libitum before blood glucose levels at 0 min were determined, followed by oral glucose administration (2 g/kg) and then measuring blood glucose levels 30, 60 and 120 min later. The area under the blood glucose curve (AUC) was calculated.

### Hyperglycemic clamp test

The hyperglycemic clamp test was performed 6 weeks after treatment as follows: mice were fasted for ~10 h with water ad libitum and then anaesthetized by intraperitoneal injection with pentobarbital sodium (80–90 mg/kg). The right jugular vein was separated and catheterized, followed by injection of a glucose solution (100 mg/kg) after a ~30 min wait. The glucose solution (10%) was continuously infused at 10–20 μL/min using a peristaltic pump. Blood glucose levels were measured every 5 min using a glucose meter, and the glucose infusion rate was adjusted to achieve a steady state of glucose concentration at 14 ± 0.5 mM. The glucose infusion rate (GIR) was calculated at the end of clamp [11].

### Statistical analysis

All the data are expressed as the mean ± standard error (SE) and analyzed by one way ANOVA (two-tailed test), except food and water consumption are only expressed as the mean with no statistical test performed. A *p*-value < 0.05 is considered to be statistically significant.

## Results

### Single administration of E2HSA lowered non-fasting blood glucose and food intake levels with a prolonged duration in diabetic KKAy mice

**Fig. 1:**
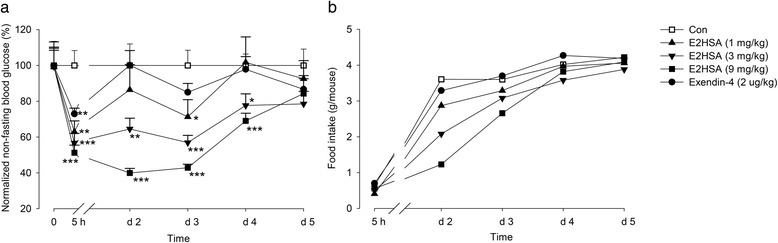
A single injection of E2HSA caused a prolonged reduction of glycemia and food intake in KKAy mice. **a** Non-fasting blood glucose levels normalized by setting blood glucose levels in the control group to 100%. Data (0) represented the non-fasting blood glucose levels when grouped before drug administration. **b** Food intake. The data for non-fasting blood glucose levels are expressed as mean ± SE, while the food intake is only expressed as the mean, *n* = 10, *** *p* < 0.001, ** *p* < 0.01, * *p* <0.05 vs. Con

Single injection of E2HSA ranging from 1 to 9 mg/kg significantly reduced non-fasting blood glucose levels in a dose-dependent manner, which continued for at least 4 days in KKAy mice. A 9 mg/kg dose of E2HSA exhibited the most potent hypoglycemic action 24 h after injection, causing a reduction of 60.1% (39.9 ± 2.6 for E2HSA vs. 100.0 ± 12.0 for Con, *p* < 0.001) (Fig. 1a). In addition, a single injection of E2HSA reduced the food intake in a dose-dependent manner 1 and 2 days after the injection, especially after the first day (Fig. 1b). A 2 μg/kg dose of exendin-4 significantly lowered the blood glucose levels by 27.1% (72.9 ± 3.2 for Exendin-4 vs. 100.0 ± 8.3 for Con, *p* < 0.01) just 5 h after injection, but the effect disappeared on the following days and had no effect on food intake.

### Repeated administrations of E2HSA restrained blood glucose variation in diabetic KKAy mice

**Fig. 2:**
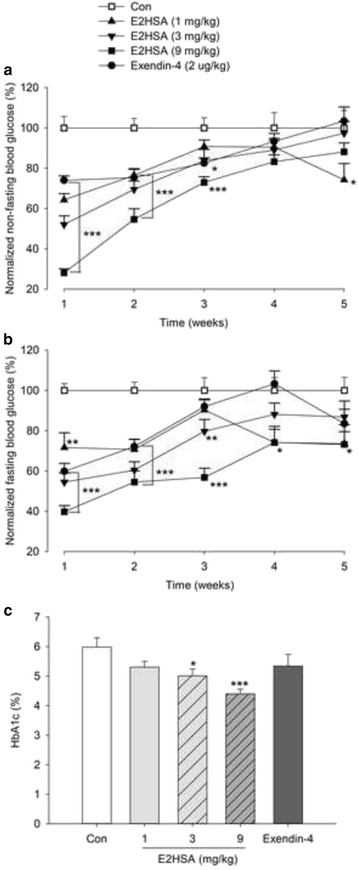
Repeated administrations of E2HSA restrained blood glucose variation in KKAy mice. **a** Non-fasting blood glucose levels were normalized by setting blood glucose levels in the control group to 100%. **b** Fasting blood glucose levels were normalized as above. **c** HbA1c levels after treatment for 6 weeks. All the data are expressed as mean ± SE, *n* = 13, *** *p* < 0.001, ** *p* < 0.01, * *p* <0.05 vs. Con

Repeated injections with E2HSA significantly reduced non-fasting blood glucose levels during the first 3 weeks of treatment, and reduced fasting blood glucose levels in a dose-dependent manner throughout the experiment, though the hypoglycemic action was decreased gradually throughout the treatment (Fig. 2a and b). Repeated injections with E2HSA at 3 and 9 mg/kg for 6 weeks decreased the HbA1c levels by 1.0% (*p* < 0.05) and 1.6% (*p* < 0.001), as well as a 0.7% reduction with 1 mg/kg, though the difference was not significant (Fig. 2c). Repeated treatments with exendin-4 (2 μg/kg) also controlled the non-fasting blood glucose levels during the first 3 weeks and the fasting blood glucose levels during the first 2 weeks, but the HbA1c levels were only decreased by 0.7%.

### Repeated administrations of E2HSA improved the impaired oral glucose tolerance in diabetic KKAy mice

**Fig. 3:**
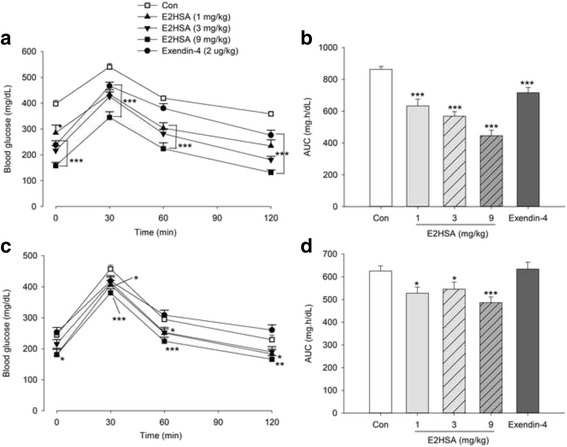
Repeated injections with E2HSA significantly improved the impaired oral glucose tolerance in KKAy mice. **a** Blood glucose levels and **b** AUC 1 week after treatment; **c** Blood glucose levels and **d** AUC 4 weeks after treatment. All the data are expressed as mean ± SE, *n* = 13, *** *p* < 0.001, ** *p* < 0.01, * *p* <0.05 vs. Con

Repeated injections with E2HSA at doses of 1, 3 and 9 mg/kg for 1 week significantly diminished increases in blood glucose levels after oral glucose administration and decreased the AUC in a dose-dependent manner (Fig. 3a and b). Along with treatment, E2HSA ranged from 1 to 9 mg/kg, controlled blood glucose variations following oral glucose gavage and lowered the AUC after 4 weeks of treatment, but the drug-effect weakened over time (Fig. 3c and d). Repeated treatment with exendin-4 (2 μg/kg) also reduced blood glucose levels and AUC following oral glucose administration 1 week after treatment, but diminished after 4 weeks following treatment.

### Repeated administrations of E2HSA corrected insulin and glucagon secretion and improved β-cell function in diabetic KKAy mice

**Fig. 4:**
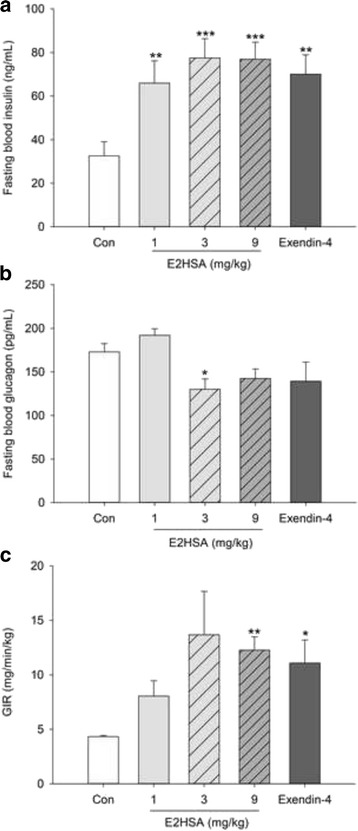
Repeated injections with E2HSA ameliorated insulin and glucagon secretion and β-cell function in KKAy mice. **a** Fasting blood insulin levels after treatment for 2 weeks, **b** Fasting blood glucagon levels after treatment for 7 weeks, **c** Glucose infusion rate (GIR). All the data are expressed as mean ± SE, *n* = 13 for A, 5–8 for B, and 3–5 for C. *** *p* < 0.001, ** *p* < 0.01, * *p* <0.05 vs. Con

Repeated injections with E2HSA significantly increased fasting blood insulin levels at doses of 1, 3 and 9 mg/kg at 2 weeks after treatment (*p* < 0.001), and decreased fasting blood glucagon levels at 3 and 9 mg/kg by 24.8% (*p* < 0.05) and 17.5% (*p* = 0.068) after 7 weeks of treatment (Fig. 4a and b). Furthermore, repeated treatments with E2HSA also increased the GIR when blood glucose levels reached a steady state of 14.0 ± 0.5 mM, with an increasing ratio of 86%, 219% and 186% for 1, 3 and 9 mg/kg, respectively (Fig. 4c). Exendin-4 (2 μg/kg) also significantly increased the fasting blood insulin levels and GIR, while decreasing the blood glucagon levels by 19.4%.

### Repeated administrations of E2HSA improved the dyslipidemia in diabetic KKAy mice

Repeated injections with E2HSA significantly lowered the fasting blood TG and TC levels in a dose-dependent manner after 1 week of treatment (Table 1), but additional file 1 showed that the effect was attenuated after 3 weeks of treatment, with a significant effect only noted at 9 mg/kg (see Additional file 1). FFA is an important intermediate product of TG. Repeated injections with E2HSA at 3 and 9 mg/kg for 2 weeks remarkably decreased the fasting blood FFA levels (Table 1). Exendin-4 (2 μg/kg) had a similar effect on fasting blood TG, TC and FFA levels with E2HSA.

**Table 1:** Repeated injections with E2HSA significantly improved dyslipidemia in diabetic KKAy mice

Group	Dose (mg/kg)	TG (mg/dL)	TC (mg/dL)	FFA (μEq/L)
Con	-	467.0 ± 58.5	193.4 ± 15.2	1295.6 ± 54.8
E2HSA	1	336.1 ± 55.1	151.3 ± 7.2***	1151.3 ± 45.1
	3	206.7 ± 14.8***	133.2 ± 7.5***	978.3 ± 32.0***
	9	159.2 ± 9.4***	105.8 ± 3.1***	878.3 ± 33.9***
Exendin-4	0.002	268.4 ± 20.4**	169.9 ± 11.4***	920.6 ± 28.5***

All of the mice were fasted for 4 h with water ad libitum before measurement. The fasting blood TG and TC levels were measured 1 week after treatment, the FFA levels were determined 2 weeks after treatment. All the data are expressed as mean ± SE, *n* = 13, *** *p* < 0.001, ** *p* < 0.01 vs. Con.

### Repeated injections with E2HSA decreased the feeding and water intake, as well as lowering body weight in diabetic KKAy mice

**Fig. 5:**
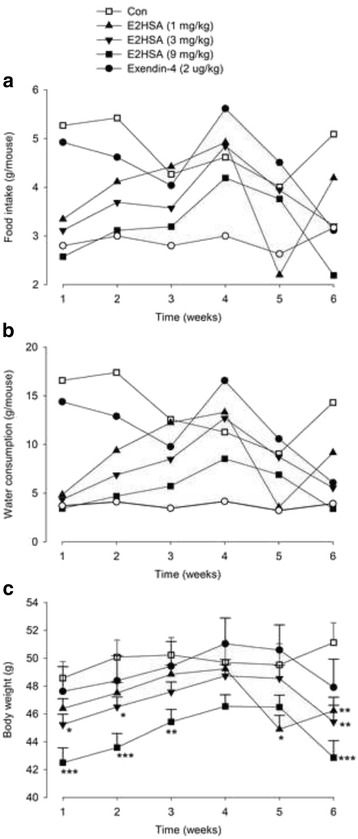
Repeated injections with E2HSA decreased food intake, water consumption and body weight in KKAy mice. **a** Food intake, **b** Water consumption, **c** Body weight. The data of food and water intake are expressed only as the mean and the body weight is measured as the mean ± SE, *n* = 13, *** *p* < 0.001, ** *p* < 0.01, * *p* <0.05 vs. Con

Repeated administrations of E2HSA significantly decreased food and water consumption in a dose-dependent manner ranging from 1–9 mg/kg, especially during the first 3 weeks, but the effect was gradually attenuated along with treatment (Fig. 5a and b). E2HSA also reduced the body weight in a dose-dependent manner in KKAy mice, which was in accordance with changes in food and water intake (Fig. 5c). Exendin-4 (2 μg/kg) also decreased the food and water consumption and reduced body weight in KKAy mice, but with weaker action when compared to E2HSA.

## Discussion

Along with the prevalence of T2DM and the trend of population senility worldwide, there is an urgent need to develop new anti-diabetic drugs. A large body of clinical research has demonstrated that GLP-1-based drugs provide excellent control of blood glucose levels without dramatic side effects [12–14]. Furthermore, GLP-1 analogs can improve β-cell function, especially in regards to insulin secretion and glucose sensitivity, while promoting β-cell survival by inhibiting apoptosis and enhancing cell proliferation [3, 15, 16]. Six GLP-1 analogs are commercially available as monotherapies for T2DM and are divided into shorting-acting exenatide and lixisenatide, long-acting liraglutide and prolonged-acting exenatide LAR, albiglutide and dulaglutide, based on the duration of action and frequency of injection [3]. Moreover, liraglutide has also been approved for treating obesity in individuals without T2DM, allowing for significant reduction in body weight [17]. Dipeptidyl peptidase-4 inhibitors, which work by increasing endogenous GLP-1 levels, are now widely-used oral agents and could potentially replace sulfonylureas in the future for significant insulinotropic and glucagonostatic actions [2]. In addition, GLP-1-based therapies have also been recommended as supplemental treatment to metformin administration when HbA1c levels failed be controlled below 7.0% with monotherapy [18], and a fixed-ratio combination of insulin degludec and liraglutide has been implemented as a therapy for T2DM [19], while another combination preparation of insulin glargine and lixisenatide is in clinical development. GLP-1-based therapies will likely predominateT2DM treatments in the future, especially for patients with poor HbA1c levels and are at risk to gain weight.

E2HSA is a long-acting GLP-1-based analog that has a half-life of ~54 h in healthy rhesus monkeys [6]. Previous studies have demonstrated that E2HSA possesses the GLP-1 receptor-dependent biological activity in vitro, indicating excellent glycemic control over prolonged durations in normal ICR mice and Sprague Dawley rats, and improved β-cell function via increased insulin secretion and β-cell mass followed chronic treatment in diabetic db/db mice [6, 9]. Therefore, E2HSA would be a novel anti-diabetic drug and is being advanced to the clinic as a candidate drug.

However, T2DM is a very complex, heterogeneous and polygenic disease, while patients with T2DM are diversified and the effects of drugs greatly vary. Thus, in order to thoroughly clarify the benefits of E2HSA and promote it to the clinic, it is necessary to evaluate its efficacies in at least three animal models that represent different pathogenesis. Db/db mouse is one of the most characteristic T2DM models induced by obesity, which results from the monogenic mutation in leptin receptor gene and becomes hyperphagic, obese (about at the age of 4 weeks), hyperinsulinemic (about at the age of 2 weeks) and insulin resistant, but later (4–8 weeks) develops hyperglycaemia and does not live longer than 8–10 months [20]. We have fully assessed the efficacy of E2HSA in db/db mice. KKAy mouse is also an obesity-induced diabetes model but with polygenic background, which originates from the Japanese KK mice by introducing the lethal yellow obese gene (A^y^) and displays severely obese, hyperglycaemic and hyperinsulinemic at about the age of 8 weeks [20]. This study aimed to evaluate the anti-diabetic effects of E2HSA in KKAy mice. Furthermore, although our previous studies showed that the hypoglycemic effect of E2HSA could last to the fifth day after single injection in normal ICR mice [9], the effect and duration in T2DM animal models are still unknown. Thus, this study also aimed to assess the action duration of E2HSA in KKAy mice, such as to supply basis for drawing up dosage regimen in the clinical experiment.

The results indicated that single injection of E2HSA decreased non-fasting blood glucose levels in diabetic KKAy mice for 4 days, suggesting the long-acting feature of E2HSA. Repeated injections of E2HSA in KKAy mice showed similar anti-diabetic benefits like that in db/db mice, including attenuating variations in fasting and non-fasting blood glucose levels, correcting the abnormal homeostasis of insulin and glucagon, and improving β-cell function, as well as ameliorating dyslipidemia, decreasing food intake and body weight, which further demonstrated the effectiveness of E2HSA.

Progressive deterioration of glycemic control is the most severe issue in diabetes treatment and plays a key role in developing related complications. HbA1c levels reflect the glycemic fluctuation within 2–3 months and have been proven to be a crucial indicator of glycemic control. Both postprandial and fasting blood glucose levels correlate with HbA1c; the former is an independent risk factor for cardiovascular disease and mortality, while the latter greatly contributes to worsened symptoms of hyperglycemia [21]. GLP-1-based analogs significantly decrease both postprandial and fasting blood glucose levels by stimulating insulin secretion and inhibiting glucagon secretion, while long-acting analogs demonstrate both greater reduction and better control of HbA1c levels with longer half-lives compared to short-acting analogs [18]. The studies both in KKAy mice and db/db mice indicated that E2HSA could significantly restrain the glycemic fluctuation through decreasing HbA1c levels.

Glycemic homeostasis is dominated by the glucose sensitivity of β cells, affecting their abilities to secrete insulin. In patients with T2DM, hyperglycemia first becomes evident by an early loss in postprandial glucose control, which mainly results from a relative defect in insulin secretion duo to diet [22]. T2DM treatments often fail due to progressive deterioration of β cells that cannot be halted, causing defects in insulin and β-cell mass loss. Our previous study found that chronic treatment with E2HSA could improve impaired oral glucose tolerance and increase both fasting and phase I blood insulin levels, while simultaneously decreasing fasting blood glucagon levels at 1 mg/kg without a dose-effect relationship in diabetic db/db mice [9]. Repeated injections of E2HSA in diabetic KKAy mice displayed similar benefits regarding glucose metabolism, but decreased fasting blood glucagon levels at a 3 mg/kg dose, indicating metabolic differences between db/db and KKAy mice, although this requires further investigation. The hyperglycemic clamp test is the gold standard for evaluating β-cell response to glucose stimulation. Repeated injection of E2HSA increased the GIR when blood glucose levels reached a steady state of 14.0 ± 0.5 mM, suggesting an improvement in β-cell sensitivity to glucose stimulation. The progressive loss of β-cell mass occurs in concordance with developing hyperglycemia, worsening health outcomes. We have demonstrated that E2HSA could enhance β-cell mass by decreasing apoptosis and increasing proliferation in db/db mice [9], while the influence of E2HSA on β-cell mass and relative mechanisms in KKAy mice are ongoing.

In addition, T2DM patients often experience dyslipidemia and obesity, which much contribute to increased cardiovascular risk. GLP-1 is known to regulate lipid metabolism and reduce body weight. Recently, GLP-1 was shown to play a crucial role in reducing hepatocyte FFA levels and may have potential in treating nonalcoholic fatty liver disease [23]. CNTO3649, a GLP-1 analog, after being continuously administered subcutaneously to APOE*3-leiden transgenic mice, fed on a high fat die, markedly reduced very low-density lipoprotein production and hepatic steatosis, in addition to improving glycemic control [24]. We previously discovered that chronic treatment with E2HSA could decrease fasting blood TG, TC and FFA levels, as well as reduce food and water intake, while also decreasing body weight in db/db mice [9]. Repeated injections with E2HSA had similar effects on lipid metabolism, food and water consumption and body weight in KKAy mice.

Although E2HSA had an enhanced duration for controlling glycemia and lipidemia in KKAy mice, this action was attenuated as the treatment progressed, which may be due to antibody production. As a recombinant protein, E2HSA contains human serum albumin and the molecular weight reaches 70 kD. Additional file 2 showed that repeated injections with E2HSA in KKAy mice significantly induced anti-drug antibody based on differences between species and large molecular weight (see Additional file 2). Many long-acting GLP-1 analogs that contain human peptides (albumin or Fc fragment of IgG) are only given once daily, based on the immune response in animals, such as CNTO736 [25] and CJC-1134-PC [26], while one analog is administered twice weekly in diabetic mice and once weekly in monkeys [27]. Therefore, E2HSA was dosed once daily both in KKAy and db/db mice, ensuring continuous coverage in the chronic experiments.

## Conclusion

In summary, acute injection of E2HSA significantly decreased non-fasting glycemia and food intake levels over a longer duration, while repeated injections restrained fluctuations in glycaemia levels, decreased HbA1c levels, corrected abnormal insulin/glucagon homeostasis and improved β-cell function, as well as ameliorating dyslipidemia and obesity in KKAy mice. Thus, E2HSA has great potential as a new GLP-1-based treatment for T2DM.

## Additional files

Additional file 1: Effects of E2HSA on fasting blood TG and TC levels after treatment for 3 weeks in diabetic KKAy mice. The results indicated that repeated injections with E2HSA only at 9 mg/kg dose significantly decreased fasting blood TG and TC levels, while the doses of 1 and 3 mg/kg had no effect. (DOCX 21 kb)

Additional file 2: The anti-drug antibody titer in KKAy mice after treatment for ~3 weeks. The results indicated that repeated injection of E2HSA at doses of 1, 3 and 9 mg/kg for 3 weeks significantly induced anti-drug antibodies, with an antibody titer ranging from 1:10,000 to 1:100,000, and the anti-drug antibody titers from mice treated with 1 mg/kg were almost 1:100,000, even one mouse was at 1:1,000,000. (DOCX 27 kb)

## Abbreviations

AUC: (Area under the blood glucose curve)
FFA: (Free fatty acid)
GIR: (Glucose infusion rate)
GLP-1: (Glucagon-like peptide − 1)
HbA1c: (Glycated hemoglobin)
T2DM: (Type 2 diabetes mellitus)
TC: (Total cholesterol)
TG: (Triglyceride)
