# Supplementary material for: The albumin-exendin-4 recombinant protein E2HSA improves glycemic control and β-cell function in spontaneous diabetic KKAy mice
Source: BMC Pharmacol Toxicol. 2017 Jun 19;18:48. doi: 10.1186/s40360-017-0143-8 (PMC5477380; doi:10.1186/s40360-017-0143-8)
Supplement: Supplementary file 1 — Effects of E2HSA on fasting blood TG and TC levels after treatment for 3 weeks in diabetic KKAy mice. The results indicated that repeated injections with E2HSA only at 9 mg/kg dose significantly decreased fasting blood TG and TC levels, while the doses of 1 and 3 mg/kg had no effect. (DOCX 21 kb) [file 40360_2017_143_MOESM1_ESM.docx]

**Additional file 1** Effects of E2HSA on fasting blood TG and TC levels after treatment for 3 weeks in diabetic KKAy mice

| **Group** | **Dose (mg/kg)** | **TG**  **(mg/dL)** |  | **TC**  **(mg/dL)** |
| --- | --- | --- | --- | --- |
| **Con** | **-** | 267.3±40.8 |  | 141.9±9.9 |
| **E2HSA** | **1** | 274.0±21.5 |  | 136.9±6.5 |
|  | **3** | 218.0±14.8 |  | 122.0±5.3 |
|  | **9** | 170.1±14.9* |  | 120.4±2.6* |
| **Exendin-4** | **0.002** | 235.9±17.2 |  | 133.9±11.6 |

All the mice were fasted for 4 h with water ad libitum before measurement. All the data are expressed as mean ± SE, n = 13, * p <0.05 vs Con.
