# Supplementary material for: The albumin-exendin-4 recombinant protein E2HSA improves glycemic control and β-cell function in spontaneous diabetic KKAy mice
Source: BMC Pharmacol Toxicol. 2017 Jun 19;18:48. doi: 10.1186/s40360-017-0143-8 (PMC5477380; doi:10.1186/s40360-017-0143-8)
Supplement: Supplementary file 2 — The anti-drug antibody titer in KKAy mice after treatment for ~3 weeks. The results indicated that repeated injection of E2HSA at doses of 1, 3 and 9 mg/kg for 3 weeks significantly induced anti-drug antibodies, with an antibody titer ranging from 1:10,000 to 1:100,000, and the anti-drug antibody titers from mice treated with 1 mg/kg were almost 1:100,000, even one mouse was at 1:1,000,000. (DOCX 27 kb) [file 40360_2017_143_MOESM2_ESM.docx]

**Additional file 2** The anti-drug antibody titer in KKAy mice after treatment for ~3 weeks

| **Animal Number** | **Anti-E2HSA (1 mg/kg) antibody titer** | | | | |
| --- | --- | --- | --- | --- | --- |
|  | **1:1000000** | **1:100000** | **1:10000** | **1:1000** | **1:100** |
| **1** | 1.9 | **5.3** | 37.5 | 108.4 | 30.3 |
| **2** | 1.1 | 1.6 | **5.2** | 24.4 | 27.6 |
| **3** | 1.2 | **2.8** | 15.6 | 59.0 | 30.3 |
| **4** | 1.1 | 1.7 | **6.1** | 27.9 | 29.4 |
| **5** | 1.7 | 1.5 | **4.1** | 19.8 | 24.8 |
| **6** | 1.3 | **2.1** | 10.3 | 49.2 | 28.0 |
| **7** | 1.2 | **2.1** | 9.4 | 43.8 | 29.0 |
| **8** | 1.7 | **4.8** | 26.9 | 102.5 | 30.3 |
| **9** | 1.8 | **6.7** | 47.9 | 127.1 | 31.1 |
| **10** | 1.4 | **3.9** | 27.7 | 92.5 | 30.1 |
| **11** | **2.1** | **8.8** | 59.9 | 125.4 | 29.8 |
| **Animal Number** | **Anti-E2HSA (3 mg/kg) antibody titer** | | | | |
|  | **1:1000000** | **1:100000** | **1:10000** | **1:1000** | **1:100** |
| **1** | 1.2 | 1.6 | **6.2** | 26.7 | 24.2 |
| **2** | 1.1 | 1.5 | **5.3** | 25.3 | 26.3 |
| **3** | 1.2 | 1.9 | **9.5** | 44.4 | 28.6 |
| **4** | 1.1 | **2.2** | 12.8 | 56.9 | 26.7 |
| **5** | 1.2 | 1.8 | **7.9** | 36.2 | 26.0 |
| **6** | 1.1 | 1.5 | **5.3** | 24.8 | 24.0 |
| **7** | 1.1 | 1.5 | **6.7** | 29.7 | 27.2 |
| **8** | 1.2 | **2.2** | 12.3 | 62.2 | 28.8 |
| **9** | 1.1 | 1.4 | **4.7** | 22.6 | 25.8 |
| **10** | 1.1 | **2.2** | 10.1 | 45.6 | 28.5 |
| **11** | 1.1 | 1.2 | **3.0** | 11.1 | 16.1 |
| **Animal Number** | **Anti-E2HSA (9 mg/kg) antibody titer** | | | | |
|  | **1:1000000** | **1:100000** | **1:10000** | **1:1000** | **1:100** |
| **1** | 1.3 | **3.3** | 20.8 | 87.6 | 32.4 |
| **2** | 1.0 | 1.6 | **4.1** | 18.1 | 24.6 |
| **3** | 1.1 | 1.7 | **6.8** | 31.4 | 29.4 |
| **4** | 1.1 | 1.2 | 1.8 | **5.6** | 11.0 |
| **5** | 1.3 | **2.3** | 10.5 | 54.8 | 30.7 |
| **6** | **2.7** | **15.4** | 110.4 | 134.2 | 31.0 |
| **7** | 1.2 | **2.0** | 7.6 | 37.9 | 30.4 |
| **8** | 1.1 | **2.1** | 7.7 | 34.5 | 29.1 |
| **9** | 1.0 | 1.8 | **5.8** | 24.6 | 26.9 |
| **10** | 1.0 | 1.5 | **4.0** | 16.4 | 23.1 |
| **11** | 1.1 | 1.2 | 1.7 | **5.2** | 9.4 |
| **Animal Number** | **Anti-Exendin-4 (2 μg/kg) antibody titer** | | | | |
|  | **1:1000000** | **1:100000** | **1:10000** | **1:1000** | **1:100** |
| **1** | 1.2 | 1.1 | 1.3 | **2.6** | 7.3 |
| **2** | 1.3 | 1.1 | 1.1 | 1.0 | 1.1 |
| **3** | 1.4 | 1.3 | **2.8** | 10.7 | 24.3 |
| **4** | 1.3 | 1.5 | **3.2** | 12.3 | 21.5 |
| **5** | 1.5 | 1.4 | 1.5 | **2.4** | 5.8 |
| **6** | 1.3 | 1.2 | 1.3 | 1.9 | **4.5** |
| **7** | 1.2 | 1.2 | 1.3 | 1.1 | 1.6 |
| **8** | 1.4 | 1.3 | 1.4 | 1.9 | **3.0** |
| **9** | 1.3 | 1.2 | 1.2 | 1.3 | 1.5 |
| **10** | 1.3 | 1.2 | 1.1 | 1.1 | 1.1 |
| **11** | 1.2 | 1.3 | 1.3 | 1.1 | 1.1 |

Serum was collected after repeated injection for about 3 weeks and diluted into 1:1,000,000, 1:100,000, 1:10,000, 1:1,000 and 1:100 with phosphate buffered saline. These diluents were added to plates that were pre-coated with E2HSA or exendin-4, followed by incubation with horseradish peroxidase labeled goat anti-mouse secondary antibody. Then the horseradish peroxidase activities were detected by adding tetramethylbenzidine and the optical density (OD) was measured at 450 nm and adjusted at 650 nm. The values of OD _treatment_/OD _control_ were calculated and the lowest dilution multiple, at which OD _treatment_/OD _control_ > 2, was defined as the anti-drug antibody titer. The numbers labeled in bold represented the anti-drug antibody which corresponded to the appropriate dilution.
